# Acute gastroenteritis associated with Rotavirus A among children less than 5 years of age in Nepal

**DOI:** 10.1186/s12879-019-4092-2

**Published:** 2019-05-22

**Authors:** Sony Shrestha, Ocean Thakali, Sunayana Raya, Laxman Shrestha, Keshab Parajuli, Jeevan Bahadhur Sherchand

**Affiliations:** 1 Department of Clinical Microbiology and Public Health Research Laboratory, Maharajgunj Medical Campus, Institute of Medicine, Tribhuvan University Teaching Hospital, Kathmandu, Nepal; 2 Department of Child Health, Institute of Medicine, Tribhuvan University Teaching Hospital, Kathmandu, Nepal

**Keywords:** Rotavirus, Genotyping, Gastroenteritis, Nepal

## Abstract

**Background:**

Rotavirus gastroenteritis is a major public health problem in Nepal. This study was conducted to obtain information associated with Rotavirus gastroenteritis and to perform genotyping of Rotavirus A.

**Methods:**

Hospital based cross sectional study was conducted from January to December 2017 among children less than 5 years of age attending Kanti Children’s Hospital and Tribhuvan University Teaching Hospital. Rotavirus A antigen detection was performed by Enzyme Linked Immunosorbent Assay (ELISA) using ProSpecT Rotavirus Microplate Assay. Rotavirus A positive strains were further confirmed by genotyping using Reverse-Transcription Polymerase Chain Reaction (RT-PCR).

**Results:**

A total of 1074 stool samples were collected, of them 770 were hospitalized, and 304 were non-hospitalized cases. Rotavirus A infection was found in 28% of children with infection rate higher in hospitalized (34%) than in non-hospitalized (14%) children. Rotavirus A detection was higher in male (31%) than in female (24%), but this was statistically not significant (*p* > 0.05). Rotavirus A positivity was higher in children of age group 0–23 months, this result was statistically not significant (*p* > 0.05) with higher frequency found in the months of November, December, January, February and March (*p* < 0.05). On the basis of molecular analysis of Rotavirus A genotyping, G12P[6] (46.39%) was found to be the predominant followed by G1P[8] (35.05%), G3P[8] (7.21%) and G1P[6] (5.15%) while 4.12% was mixed infection and 1.03% was partially typed (*p* < 0.05).

**Conclusion:**

Rotavirus A infection occurred throughout the year, but the infection was significantly higher during the month of March. The higher frequency of rotavirus infection was observed among children of age group 0–23 months; however this was not found to be statistically significant. In this study, G12P[6] is predominant genotype observed. The results of genotyping are essential for the introduction of Rotavirus vaccine in Nepal.

**Electronic supplementary material:**

The online version of this article (10.1186/s12879-019-4092-2) contains supplementary material, which is available to authorized users.

## Background

Acute gastroenteritis (AGE) is an extremely common illness among infants and children worldwide [1]. It is an inflammation of the gastrointestinal tract characterized by diarrhea and symptoms of gastric irritation (e.g nausea, vomiting, and epigastric pain) caused by microbial agents [2, 3]. Diarrhea is defined by World Health Organization (WHO) as having 3 or more loose or liquid stools per day or as having more stools than in normal for that person [4]. Diarrheal diseases are associated with an estimated 1.3 million deaths annually, with most occurring in resource-limited countries; up to 25% of deaths in young children living in Africa and south-east Asia are attributable to AGE [5].

Diarrhea can be caused by a wide range of virus, bacteria and parasites. In both developed and developing countries, viruses are the leading cause of acute diarrhea [6]. Among all viral diarrheal agents, Rotavirus is a major cause of AGE which results in nearly 200,000 deaths annually in children younger than 5 years [7, 8]. A systematic review of Rotavirus A gastroenteritis in children less than five years of age from Asia, found Rotavirus A to be associated with approximately 145,000 deaths every year [8]. By the age of five years, nearly every child has been infected at least once and reinfection is mild [9].

Nepal being a developing country, diarrheal diseases are major problem. Precise data on childhood mortality associated with diarrheal diseases in Nepal is not available. But in 2016/17, 1,184,120 cases of Diarrhoea were reported of which 0.44% suffered from severe dehydration (increased from 0.2% the previous year). The national incidence of diarrhoea per 1000 under-5 year olds decreased from 422/1000 in 2015/16 to 400/1000 in fiscal year 2016/17 [10].

Symptoms of Rotavirus A disease often start within 1–3 days of infection with vomiting followed by 4–8 days of profuse diarrhoea. Rotavirus A replicates in the cytoplasm of mature enterocytes lining the tips of intestinal villi, preventing uptake of nutrients and causing severe diarrhea that can be fatal if left untreated. Rotavirus A sheds in high concentration in the stool and spreads by fecal-oral route before two days and after ten days of onset [11].

There are differences in the distribution of Rotavirus A infections according to age in developing and developed countries. In the former, the highest rates occur during the first year of life. However, in developed countries, peak rates occur in the second year of life. This could emphasize the need of Rotavirus A vaccine being applied earlier in life to children in developing countries [12]. It is generally believed that serotype-specific immunity plays a role in protection against disease, so the epidemiology of circulating genotype of Rotavirus A forms a critical knowledge base for the development and implementation of Rotavirus vaccines [13].

So, this study was conducted to obtain more information about Rotavirus A gastroenteritis which is important for health care workers and policymakers. Such information can be helpful to improve the diagnosis and treatment of infection and also will be useful to provide information for future projection and evaluation of child health diarrheal disease and future vaccine implementation.

### Research questions

- Is there increasing trend in rotavirus gastroenterititis among Children in Nepal?
- Is there any significant relation between AGE and the age, gender and month of sample collection?

## Methods

The study was carried out in Deparment of Clinical Microbiology, Public Health Research Laboratory, Institute of Medicine, Tribhuvan University Teaching Hospital (TUTH), Maharajgunj, Kathmandu. Written informed consent was requested from children’s parents/guardians from all eligible cases. Stool samples were requested from the children under 5 years of age visiting Kanti Children’s Hospital and TUTH, Kathmandu, with acute diarrhoea in the period between January to December 2017 and whose parents/guardians provided consent to participate in the study.

### Inclusion criteria

Case-patients were eligible for enrollment in the study if they met the following criteria:

1. All children under 5 years who presented at the study site/hospital for treatment of diarrheal illness with or without intake of medications.
2. Has acute diarrhea with duration of ≤14 days.
3. Admission to the diarrheal treatment unit (IPW) and hospital catchment area.

### Exclusion criteria

Cases were excluded if they met any of the following criteria:

1. Children with diarrhea occurring after hospital admission- that is children admitted with separate diagnosis (non-diarrhea) initially and later developing diarrhea at any time during admission.
2. Children presenting or diagnosed to have chronic or persistent diarrhea (diarrhea that last for more than 2 weeks).

### Sample collection, storage and testing

From each participating child, clinical data were obtained and the stool sample was collected in a sterile container. The collected stool samples were preserved at -20 °C until they were analysed. Rotavirus A infection was tested using an antigen detection test by ELISA (ProSpecTM Rotavirus Microplate Assay, Oxoid) according to the instructions of the manufacture. Then the samples were stored at -80 °C for genotyping. Subsequently random 100 Rotavirus A antigen positive samples were subjected to genotyping by RT-PCR.

For molecular typing, genomic RNA was extracted using the QIAamp viral RNA mini kit (Qiagen), according to the manufacturer’s instructions. VP7 (G) and VP4 (P) genotypes were detected by RT-PCR according to methods described previously [13–15].

For Rotavirus G genotyping, the VP7 gene was amplified by RT-PCR with VP7/F and VP7/R primers (Table 1). For Rotavirus P genotyping, the VP4 gene was amplified by RT-PCR with con-2 and con-3 primers (Table 2).

**Table 1:**
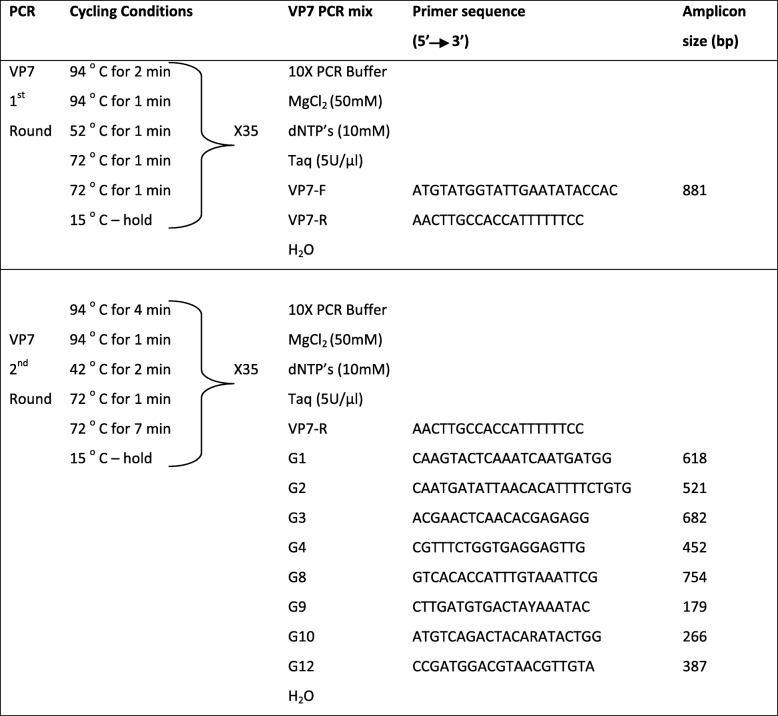
PCR primers and cycling conditions used for VP7 genotyping of Rotavirus A strains

**Table 2:**
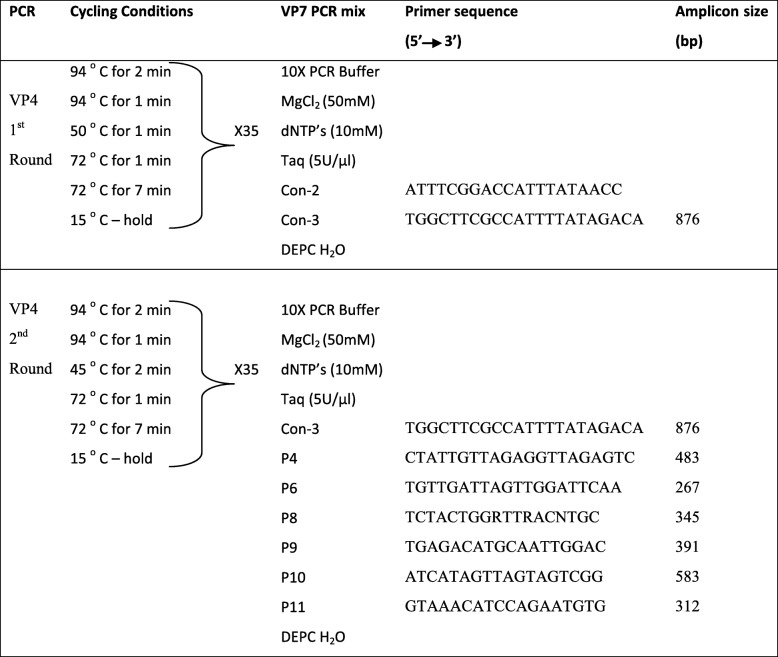
PCR primers and cycling conditions used for VP4 genotyping of Rotavirus A strains

### Data interpretation

#### Gel electrophoresis and documentation

Identification of Rotavirus genotypes were done on the basis of PCR amplicon size by using gel electrophoresis. Preparation of 2% agarose gel was done by weighing 2 g of agarose in 100 ml of 1X Tris-boric acid EDTA (TBE) and was stained with ethidium bromide (0.5 mg/ml). The gel was poured onto set electrophoresis tray and PCR amplicons were resolved at 100 V for 2 h. Images were photographed under UV light using a gel documentation system.

#### Validity and reliability

For ELISA test, inactivated bovine Rotavirus mixed with buffer was used as positive control and tris buffered saline solution as negative control. For genotyping PCR assays we used genotype G1P[8] as a positive control.

#### Analysis

Data entry was done in Excel and analyzed using SPSS (Version 16). *P* values were calculated using Chi- square test and value < 0.05 were considered statistically significant.

## Results

A total of 1074 stool samples examined for Rotavirus A using ELISA, 302/1074 (28%) samples were found Rotavirus A positive with infection rate being higher in hospitalized (i.e. all hospital admitted cases) 261/770 (34%) than in non-hospitalized (cases seen in OPD) 41/304 (14%) children, this observation was statistically significant (*p* < 0.05). Rotavirus A detection was higher in male (197/642 = 31%) than in female (105/432 = 28%) (*p* > 0.05).

### Distribution of rotavirus infection on the basis of age

Though Rotavirus A infection was seen highest in children of age group 0–11 months (30%) followed by 12–23, this result is statistically not significant (27%) (*p* > 0.05) as shown in Table 3.

**Table 3:** Age distribution of Rotavirus A infection

Age group (month)	Total number of cases	Total Rotavirus positive cases (%)	Total hospitalized cases	Rotavirus positive among hospitalized cases (%)	Total non-hospitalized cases	Rotavirus positive among Non-hospitalized cases (%)
0–11	646	196 (30)	455	162 (36)	191	34 (18)
12–23	287	77 (27)	209	70 (34)	78	7 (9)
24–59	141	29 (21)	106	29 (27)	35	0
Total	1074	302 (28)	770	261 (34)	304	41 (14)

### Monthly occurrence of rotavirus a diarrhea

**Fig. 1:**
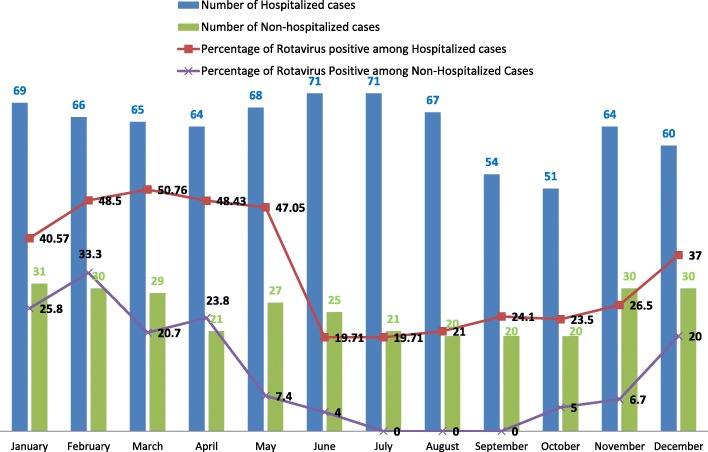
Monthly occurrence of Rotavirus diarrhoea

In this study, isolation of Rotavirus A was found to be highest in the month of March followed by February, this finding was statistically significant (*p* < 0.05) as shown in Fig. 1

### Clinical symptoms and rotavirus detection among diarrheal children

The most common clinical symptoms among the children enrolled were dehydration (some 92% and severe 8%), fever (25%), vomiting (36%), nausea (43%) and abdominal pain (100%). Rotavirus A infection among cases with some dehydration is 26% and severe dehydration 47%, whereas infection rate in children with fever, vomiting, nausea and abdominal pain is 27, 40, 29 and 28% respectively.

### Genotypic distribution of rotavirus a

**Fig. 2:**
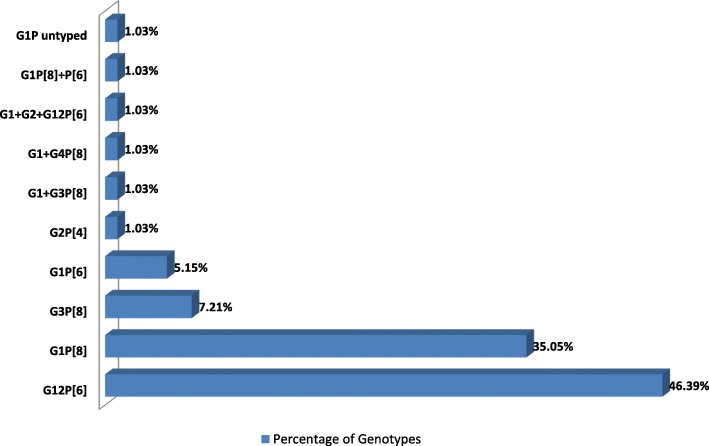
Genotypic distribution of Rotavirus A

On the basis of genotyping results of Rotavirus A, G12P[6] (46.39%) was found to be the predominant genotype followed by G1P[8] (35.05%), G3P[8] (7.21%) and G1P[6] (5.15%) while 1.03% was partially typed, this finding was statistically significant (*P* < 0.05) as elucidate in Fig. 2.

**Fig. 3:**
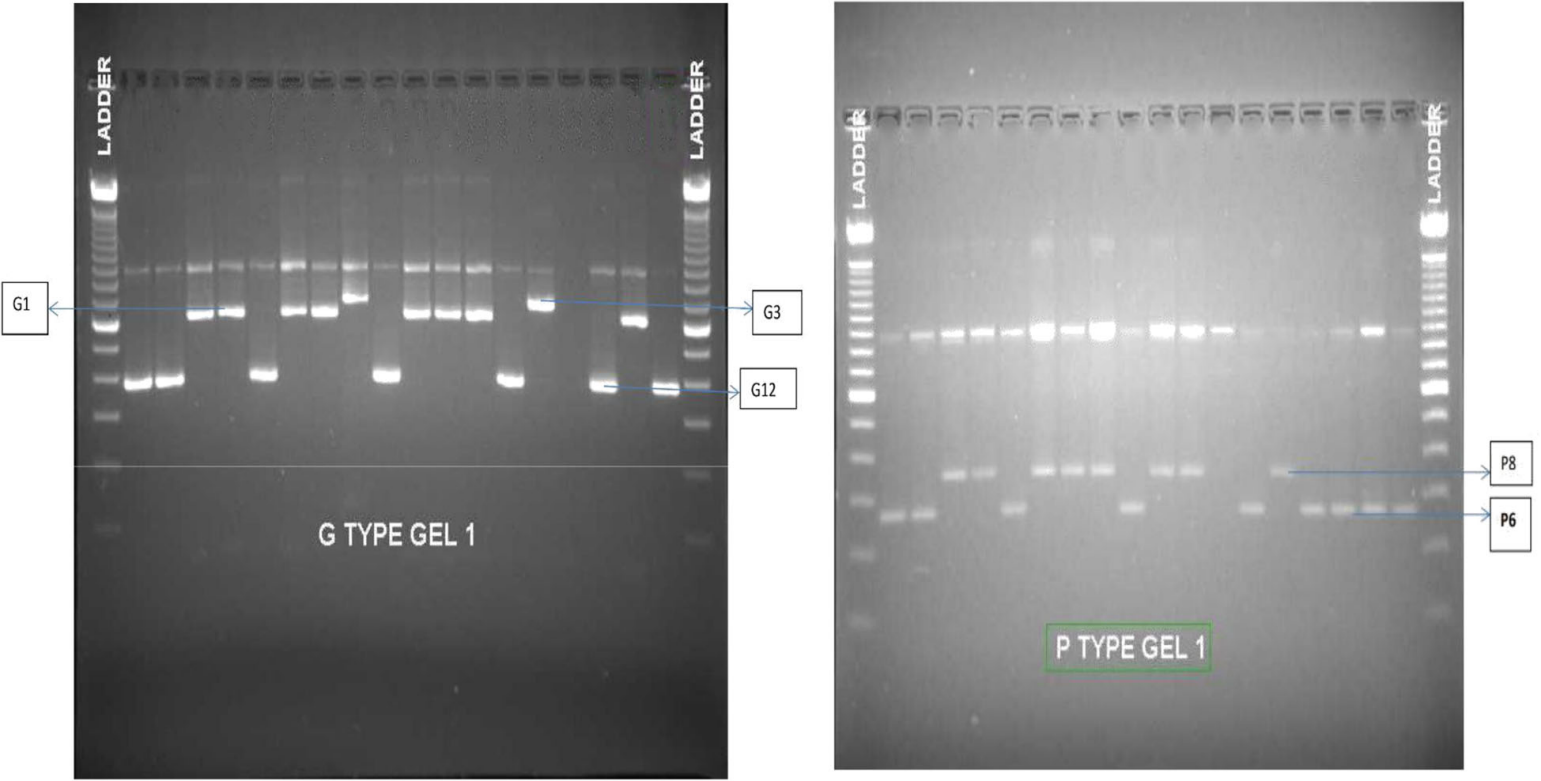
Gel run of G-type and P-type PCR analysis

The genotype was determined on the basis of gel electrophoresis of the product which differed according to the base pairs as shown in Fig. 3.

## Discussion

The present study revealed that Rotavirus A infection was 28%. This study is similar to other studies conducted by Sherchand et al. (35.4%) [16], Dhital et al. (22.9%) [17], Ansari et al. (25.9%) [18], Pun et al. (23.5%) [19], Saeed et al. (22%) [20] and Akran et al. (28.9%) [21] while Reither et al. (55%) [22] reported slightly higher positivity rate. Although, improvements in nutrition hygiene, increased awareness among caretakers and the use of oral rehydration therapy contributed to declining the incidence of diarrheal diseases over the past years in Nepal, this result revealed that Rotavirus A infection remained high among diarrheal children [19].

In this study, the infection rate of Rotavirus A is higher in hospitalized 261 (34%) than in non-hospitalized 41 (14%) children, this observation was statically significant (*p* < 0.05). This is in accordance with other studies [13, 14, 16, 23, 24]. Gender distribution of diarrheal cases showed higher prevalence in male (30.69%) than in female (24.31%). Although some other studies also observed a higher prevalence of diarrhea among male [18, 25], but prevalence of Rotavirus infection among gender was not found to be statically significant in this study. Prevalence of Rotavirus A among younger age group was statistically not significant. This may be due to sample collection bias. In the case of younger ones, parents are more concern and hence, they visit the hospital immediately whereas in elder ones, they don’t visit the hospital unless it become severe. Our result is closer to the results of Sherchand et al. [23], Ahmed et al. [26] and Johargy et al. [27] . Thus, vaccination at an early age can be beneficial to prevent majority cases of diseases [17].

Rotavirus A infection occurred all year round but peaked during the month of March followed by February that is during the dry weather, this result was statistically significant (*p* < 0.05). This finding is in accordance with other studies reported by Sherchand et al. [23], Pun et al. [19] and Dhital et al. [17].

In our study, abdominal pain was the most common clinical symptom among 1074 children with acute diarrhea. Whereas, the degree of dehydration among enrolled cases was more likely to be some to severe.

On the basis of molecular analysis of Rotavirus A, G12P[6] (46.39%) was found to be the most common genotype followed by G1P[8] (35.05%), G3P[8] (7.21%) and G1P[6] (5.15%) while 4.12% was mixed infection and 1.03% was partially type. The predominance of G12P[6] is in accordance with other studies of Sherchand et al. (45.7% in 2009) and (28.47% in 2010) [23], Ansari et al. (46.4% in 2011), Sherchand et al. (48% in 2012) [16]. Approximately 90% of all human rotaviral diarrhea is due to 5 G-P combinations (G1P[8], G2P [4], G3P[8], G4P[8]) and G9P[8]) globally [17]. G12 is also becoming important in human diarrheal disease as it is recognized as an emerging genotype [23, 28]. It has been reported that unusual G and P combinations constitute more than 14% of reported Rotavirus A isolates from Asia, 27% from Africa, 11% from South America, 5% from North America, 1.4% from Europe, and 0.1% from Australia [29].

After Rotavirus A vaccine introduction there is fluctuation in the genotype distribution annually and geographically [30]. In Nepal, still Rotavirus A vaccination has not been incorporated in the national immunization schedule, but due to high burden of Rotavirus A infection vaccination is necessary. After execution of vaccination program decrease in the burden of diarrhea can be expected from the experience of countries like USA, Finland, Belgium, Brazil, Venezuela and Mexico where Rotavirus A vaccination has been adopted [17].

### Limitation of the study

This study was limited only to patients of acute diarrhea visiting hospital in Kathmandu and time framework for the study was only 1 year.

## Conclusion

The present study indicates that Rotavirus A was the cause of 28% of the diarrheal cases studied and that Rotavirus is major problem in children under 5 years of age. Molecular analysis revealed that G12P[6] were the major genotype followed by G1P[8], G3P[8] and G1P[6] causing Rotavirus diarrhea. High prevalence of group A Rotavirus infection in children with diarrhoea and also the determination of circulating Rotavirus A genotypes provides useful data for formulating new and more effective vaccines, especially for infants. As these findings do not represent the complete scenario nationwide, further analysis is necessary to determine the prevalence of rotaviral infection in Nepal.

## Additional file

Additional file 1: Rotavirus dataset, 2017(the data generated during this study, includes the data generated like month of hospital visit, number of diarrhea, vomiting, ELISA results, genotyping results in excel sheet.) (XLSX 157 kb)

## Abbreviations

AGE: Acute gastroenteritis
ELISA: Enzyme Linked Immunosorbent Assay
IPW: In-patient ward
RT-PCR: Reverse-Transcription Polymerase Chain Reaction
TBE: Tris-boric acid EDTA
TUTH: Tribhuvan University Teaching Hospital
WHO: World Health Organization
